# Fractional Microneedling Radiofrequency for Hidradenitis Suppurativa: A Real‐World Retrospective Study Demonstrating Clinical Efficacy and Safety Across Diverse Anatomical Sites

**DOI:** 10.1111/jocd.70748

**Published:** 2026-02-24

**Authors:** Ari Safir, Eyal Taleb, Daniella Berzin, Manny Arieli, Aspasia Liassidou, Waseem Shehadeh, Ariela Hafner, Ofir Artzi

**Affiliations:** ^1^ Division of Dermatology Tel Aviv Sourasky Medical Center Tel Aviv Israel; ^2^ Faculty of Medical & Health Sciences Tel Aviv University Tel Aviv Israel

## Abstract

**Background:**

Hidradenitis suppurativa (HS) remains a therapeutically challenging disease despite expanding research and evolving systemic treatments. Energy‐based modalities, such as fractional microneedling radiofrequency (FMR), are being increasingly explored as novel treatment options.

**Aim:**

To evaluate the real‐world efficacy and safety of Morpheus8‐based FMR treatment in patients with HS.

**Materials and Methods:**

This retrospective analysis included 25 HS patients treated at a tertiary dermatology center. Seventeen patients who completed ≥ 2 FMR sessions were included in the efficacy analysis. Outcomes were assessed by the International Hidradenitis Suppurativa Severity Score System (IHS4) and IHS4‐55 (≥ 55% reduction). High‐frequency ultrasound (HFUS) was used in selected cases to assess treatment response and inflammatory changes.

**Results:**

Sixteen of the 17 patients (94.1%) evaluated for efficacy showed clinical improvement, and nine (52.9%) achieved an IHS4‐55 response. The mean IHS4 reduction was 4.6 ± 2.5. The highest response rates were observed in the face, chest, and gluteal regions, while groin and thigh showed the lowest. HFUS confirmed a reduction in inflammation. Treatment was generally well tolerated, with pain cited as the most common reason for discontinuation in 3 of 25 patients (12%).

**Conclusion:**

FMR appears to be a safe and effective treatment option for patients with moderate‐to‐severe HS, including those with refractory disease and lesions in anatomically challenging areas. Clinical outcomes in our cohort were comparable to those reported for advanced systemic agents and other technology‐based interventions. These findings support FMR's potential role in personalized HS management ‐ either as a standalone intervention or in combination with systemic or procedural treatments.

## 1 Introduction

Hidradenitis suppurativa (HS) is a chronic, relapsing inflammatory skin disorder that affects follicle‐bearing intertriginous areas. It is characterized by painful nodules, abscesses, draining tunnels, and disfiguring scars, significantly impairing quality of life and associated with high rates of depression and suicide [1, 2]. Therapeutic options for HS have recently expanded, with systemic agents, including biologics, having become more widely available, and technological interventions, such as hair removal lasers, CO_2_ lasers for deroofing, and other ablative modalities, being increasingly incorporated into treatment algorithms [3, 4]. Despite these advancements, a substantial proportion of patients remain undertreated or refractory to these therapies, underscoring the need for additional effective, repeatable, and minimally invasive alternatives.

Fractional microneedling radiofrequency (FMR), a modality widely used in esthetic dermatology, has shown promising results in the treatment of inflammatory dermatoses and scarring disorders [5, 6]. Preliminary studies have demonstrated that FMR may downregulate key cytokines implicated in HS pathogenesis, including TNF‐α, IL‐17, and TGF‐β, while promoting dermal remodeling [7]. Nevertheless, real‐world evidence of its clinical utility in HS, especially across diverse anatomical regions, remain limited.

This study was conducted at a tertiary dermatology center with a designated HS unit offering a wide spectrum of medical, surgical, and device‐based therapies. Between 2022 and 2025, both male and female patients with variable disease durations and severities were treated by FMR across multiple anatomical regions, including the groin, gluteal area, chest, face, and intermammary folds. We now aim to evaluate the safety and efficacy of FMR in a real‐world HS population of patients with refractory disease or lesions located in anatomically challenging sites.

## 2 Methods

### 2.1 Study Design and Setting

We conducted a retrospective observational study at a dermatology division within a tertiary medical center, reviewing the medical records of patients treated with FMR by means of the Morpheus8 device (InMode Ltd.) between June 2022 and April 2025. The study protocol was approved by the local institutional review board. Patients provided written informed consent for the use of their anonymized photographs for research and publication purposes.

### 2.2 Patient Referral for FMR Treatment

Patients were referred for treatment with FMR based on the presence of active HS with suboptimal response to past or ongoing systemic, surgical, or technological therapies and fulfillment of one or more of the following criteria:

(1) Limitations associated with escalating systemic therapy, including patient refusal, medical contraindications, or ineligibility for reimbursement by their health maintenance organization (HMO).

(2) Prior failure or ineligibility for other energy‐based modalities (e.g., long‐pulsed Nd:YAG laser) due to anatomical or clinical factors.

(3) Anatomical lesion distribution or overall disease burden precluding their suitability for surgical excision or deroofing.

FMR was offered to these patients as a complementary or alternative therapeutic option. Although not randomized, this criteria‐based recruitment strategy reflects real‐world clinical decision‐making and resource allocation.

### 2.3 Treatment Reimbursement Authorization Process

Radiofrequency (RF)‐based therapies have recently been incorporated into European treatment guidelines as an optional modality for HS [8]. Patients whose requests for reimbursement by their HMOs were denied could elect to self‐fund their treatment.

### 2.4 Patient Selection

We included patients aged 16 years or older with a clinical diagnosis of HS who underwent treatment with FMR with the Morpheus8 device and for whom complete baseline and follow‐up clinical data were available.

### 2.5 Treatment Protocol

The treatment was performed by means of Morpheus8 Body 3D or 40‐pin microneedle tips delivering bipolar RF energy at variable depths (typically 4–7 mm for HS lesions), adjusted according to anatomical location and severity. Energy levels ranged from medium to high (20–60), using multiple stacks at multiple depths. Treatment was performed under topical anesthesia or, in selected cases, under tumescent anesthesia due to procedural pain. Treatment sessions were typically spaced 6–8 weeks apart with the total number of sessions determined by clinical response. Treatment sessions were gradually spaced further apart as disease control improved, with eventual discontinuation following prolonged clinical stability.

### 2.6 Outcome Measures

Objective and subjective outcomes were derived from standardized scores and clinical documentation as follows: International Hidradenitis Suppurativa Severity Score System (IHS4): Used to quantify disease activity at baseline and post‐treatment [9]; IHS4‐55 response: Defined as ≥ 55% reduction in IHS4 from baseline [10]; high‐frequency ultrasound (HFUS) with high resolution multifrequency 4–18 MHz linear array transducer and 4–15 MHz compact linear array transducer (Affiniti70s Philips, Philips medical systems, Bothell Wash., USA) was performed in a subset of patients at baseline and during follow‐up to objectively assess treatment response. Evaluation of echogenicity, thickness, presence of typical HS sonographic lesions, cysts, edema, and vascularity by color Doppler were included [11, 12, 13]. For efficacy analysis, only patients who completed two or more treatment sessions were included based on a predefined criterion that at least two sessions were necessary to evaluate clinical response.

#### 2.6.1 Safety Monitoring

All adverse events, including clinical worsening, erythema, edema, infection, or delayed healing, were recorded. Serious adverse events were defined as those requiring hospitalization or resulting in permanent tissue damage.

### 2.7 Data Analysis

Descriptive statistics were used to summarize patient demographics, anatomical treatment sites, and treatment parameters. Clinical efficacy was evaluated by pre‐ and post‐treatment IHS4 scores, which were analyzed with paired statistical comparisons. The Wilcoxon signed‐rank test was used to assess changes in IHS4, given the nonparametric distribution of the data. Subgroup comparisons were not powered for statistical inference in this exploratory retrospective analysis. Visualizations included paired dot plots, boxplots, and stratified comparisons by anatomical region. All data were anonymized and stored securely in compliance with institutional privacy protocols. Statistical analyses were performed using R (version 4.2.2) via the R2 platform (https://r2.amc.nl).

#### 2.7.1 Patient‐Reported Outcomes

The structured patient‐reported outcome measure that was administered post‐treatment had been adapted from the Patient Global Impression of Change (PGIC) framework. This included a multidomain questionnaire assessing global improvement in HS symptoms, pain levels in the treated areas, frequency of disease flares, impact upon daily functioning, and overall satisfaction with treatment. Responses were graded on a seven‐point Likert scale ranging from “very much improved” to “very much worse,” with additional multiple‐choice and open‐text items to capture qualitative feedback. Patients who discontinued follow‐up were also contacted by telephone to assess their reasons for discontinuation and to record retrospective impressions of treatment efficacy and tolerability. The fully translated questionnaire is available in Supporting Information. (Table SS1).

## 3 Results

### 3.1 Study Cohort, Treatment Areas and Lesion Characteristics

A total of 25 patients diagnosed with HS were included in the study, comprising 11 males (44%) and 14 females (56%), ranging in age from 16 to 59 years (mean 31.7). Seventeen patients (68%) were current or former smokers. Common comorbidities included acne (12.0%), pilonidal sinus (12.0%), and polycystic ovary syndrome (12.0%), consistent with known associations in HS (Table 1).

**TABLE 1:** Baseline characteristics of 25 patients with hidradenitis suppurativa treated with fractional microneedling radiofrequency, stratified by number of treatment sessions (< 2 vs. ≥ 2).

Characteristics by number of treatment sessions	Total	< 2	≥ 2
Total patients, *n*	25	8	17
Age, mean (range), years	31.7 (16–59)	25.5 (17–35)^b^	34.6 (29–40)^b^
Female, *n*	14 (56.0%)	5 (62.5%)	9 (52.9%)
Smoking history, *n*	17 (68.0%)	5 (62.5%)	12 (70.6%)
Comorbidities, *n*			
Acne	3 (12.0%)	0	3 (17.6%)
Pilonidal sinus	3 (12.0%)	1	2 (11.8%)
Polycystic ovary syndrome	3 (12.0%)	1	2 (11.8%)
Disease duration. years, mean (SD), (range)	12.2 (7.5), (1.0–35.0)	9.2 (2.8), (1.0–16.0)	13.5 (7.8), (4.0–35.0)
Hurley stage, *n*			
I	14 (56.0%)	5 (62.5%)	9 (52.9%)
II	10 (40.0%)	3 (37.5%)	7 (41.2%)
III	1 (4.0%)	0	1 (5.9%)
IHS4 severity, *n* ^a^			
Mild	4 (16.7%)	3 (42.9%)^b^	1 (5.9%)^b^
Moderate	16 (66.7%)	4 (57.1%)^b^	12 (70.6%)^b^
Severe	4 (16.7%)	0^b^	4 (23.5%)^b^
Prior treatments, *n*			
Topical	21 (84.0%)	5 (62.5%)	16 (94.1%)
Systemic	22 (88.0%)	5 (62.5%)	17 (100%)
Surgical	8 (32.0%)	2 (25%)	6 (35.3%)

^a^ IHS4 severity scores were available for 24 of 25 patients.

^b^ Statistically significant differences were observed between patients who discontinued early (< 2 sessions) and those who completed ≥ 2 sessions: patients who continued were older (*p* = 0.003) and had more severe baseline IHS4 scores (*p* = 0.01).

The mean disease duration was approximately 12.7 years, underscoring the chronic and treatment‐refractory nature of this cohort. At baseline, 56% of patients had Hurley stage I, 40% stage II, and 4% stage III disease. Based on IHS4 scoring, 16.7% had mild disease, 66.70% had moderate disease, and 16.7% had severe disease.

Most patients had been previously treated with topical (84%), systemic (88%), and surgical interventions (32%), and 16% had received biologic therapies. Among those with prior surgical management, seven patients (30.4%) had undergone incision and drainage procedures, eight (34.8%) had been treated with deroofing techniques, and three (13.0%) had undergone wide surgical excision, reflecting the chronicity, severity, and treatment‐refractory nature of the cohort. Lesion characteristics included inflammatory nodules (60%), tunnels (36%), and cysts (36%), of which 44% were in combination. The patients received a median of three FMR treatments (range 1–6; mean 3.3 ± 1.95) which were applied to a range of anatomical regions, most commonly the groin (8/25), gluteal area (7/25), face (7/25), chest (6/25), and, less frequently, the nape (1/25) and back (1/25), underscoring the anatomical diversity and complexity of HS within the cohort.

FMR parameters were tailored to lesion depth and anatomical location, typically ranging from 3 to 7 mm in penetration depth and 30–60 units of energy delivered through stacked passes in fixed or cycling modes. This individualized approach enabled effective targeting of deep‐seated lesions while minimizing discomfort in more sensitive regions (see Table S2 for full parameter details).

### 3.2 Clinical Response

Only patients who received at least two treatment sessions were included in the efficacy analysis, and 17 of the original 25 treated patients met this criterion. Comparisons between patients who continued the treatment and those who discontinued early revealed differences in baseline age and disease severity: those who continued were older (mean 34.6 vs. 25.5 years; *p* = 0.003) and had higher baseline IHS4 scores (*p* = 0.01) (Table 1). Groin and thigh involvement were more common among early discontinuers, although this trend did not reach a level of significance. No other significant differences in demographic or clinical characteristics were observed between these two groups.

**FIGURE 1:**
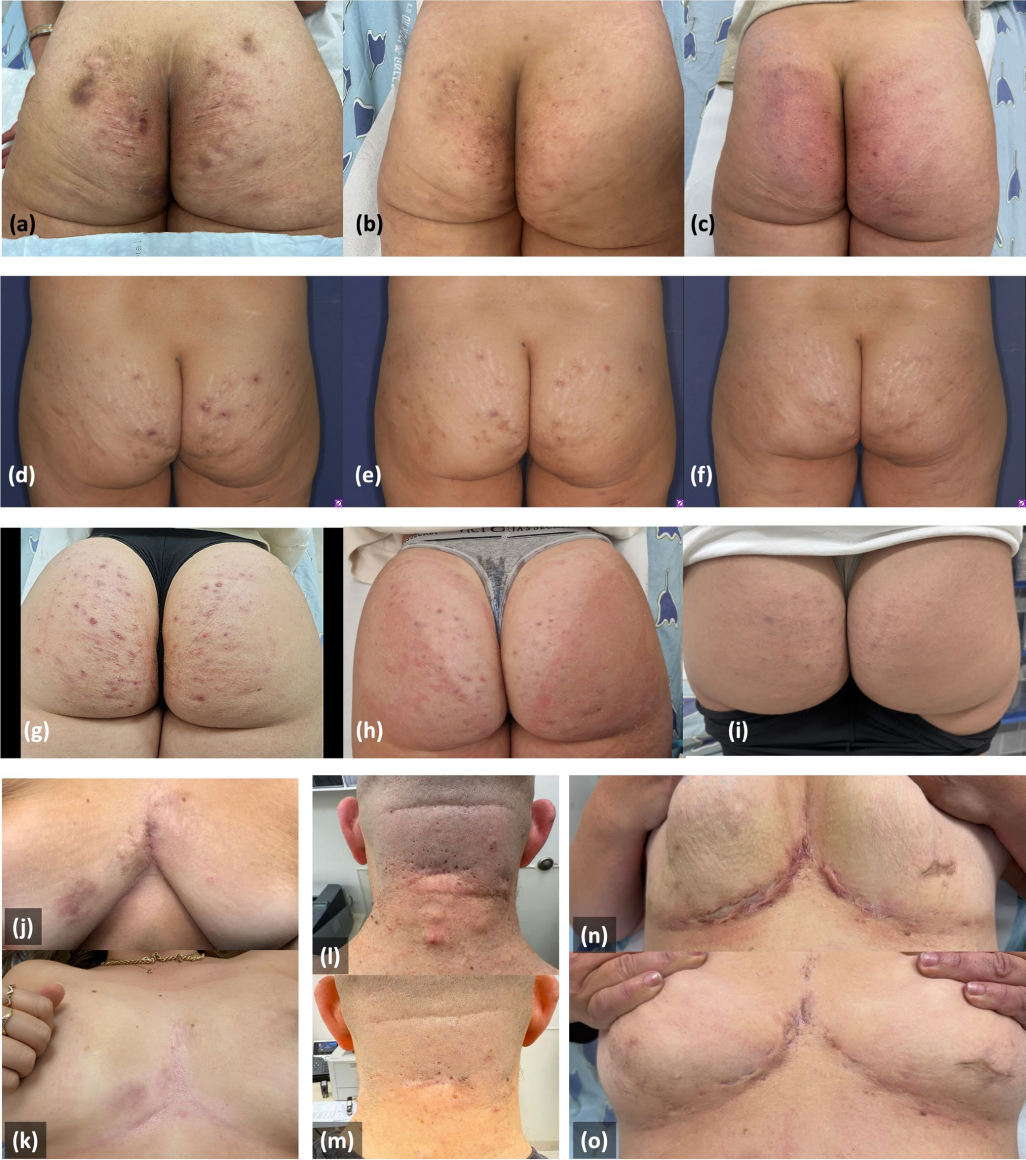
Clinical improvement following fractional microneedling radiofrequency treatment. Representative before and after photographs of patients with hidradenitis suppurativa (HS) treated with fractional microneedling radiofrequency (FMR). The images illustrate clinical responses across multiple treatment sessions, with visible reductions in inflammation, nodules, and scarring. Progressive improvements in skin texture, pigmentation, and overall lesion resolution are noted over time. (a–i) Gluteal HS lesions. Marked improvement is shown in three patients: (a–c) after three sessions, (d–f) after four sessions, and (g–i) with progression after two (h) and three (i) sessions. (j–k) Intermammary fold lesions showing resolution of active disease, reduction in post‐inflammatory changes, and tunnel activity. (l–m) Nuchal HS lesions demonstrating reduced inflammation and active disease. (n–o) Bilateral inframammary and chest wall involvement showing improvement in inflammatory lesions.

**FIGURE 2:**
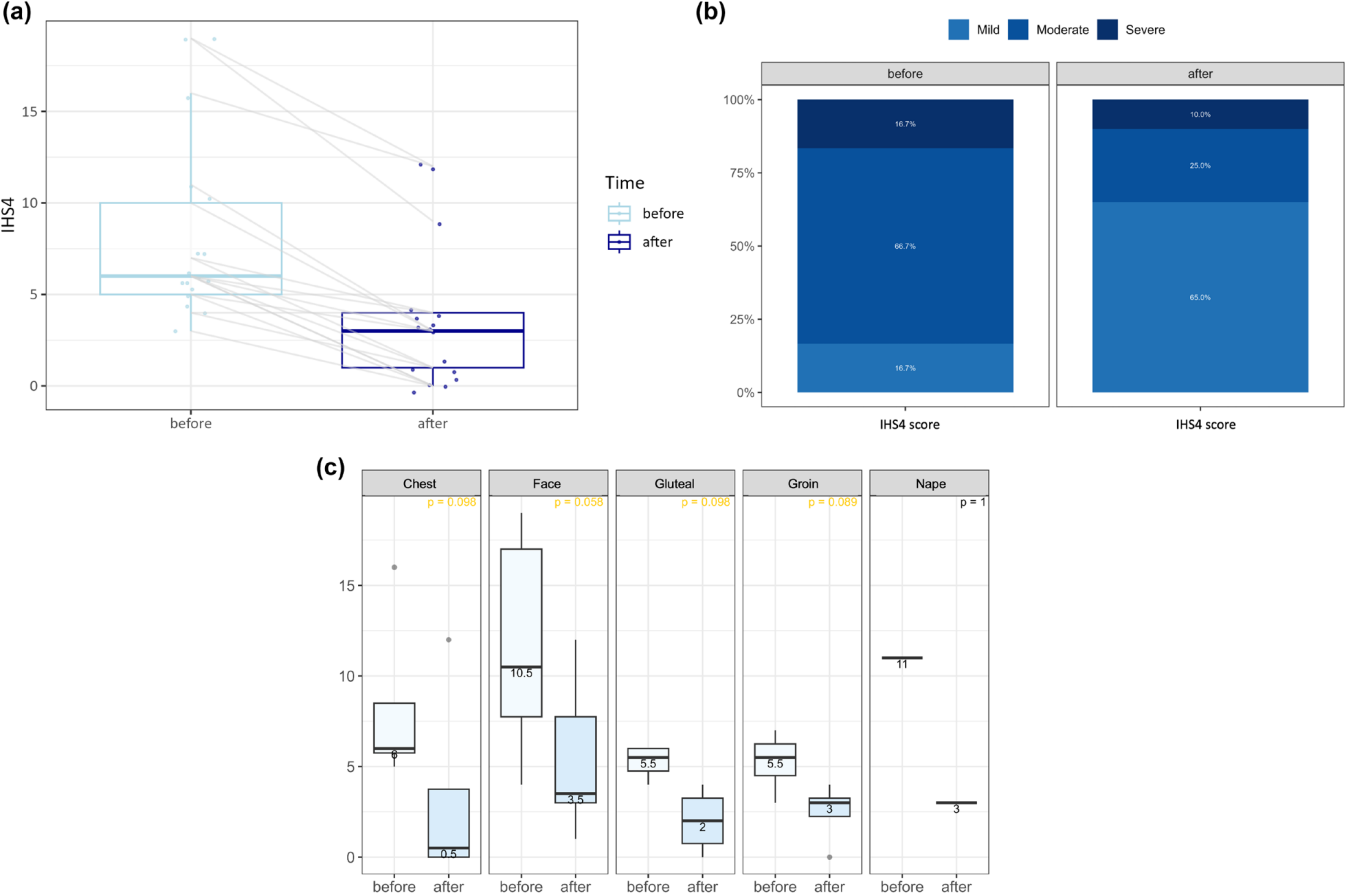
Improvement in International Hidradenitis Suppurativa Severity Score System (IHS4) severity following fractional microneedling radiofrequency treatment. (a) Paired boxplots demonstrating individual and overall changes in IHS4 values before and after treatment among evaluable patients (*n* = 17). Each gray line represents one patient. A reduction in median IHS4 is observed following treatment. (b) Stacked bar chart showing the distribution of disease severity (mild, moderate, severe) based upon IHS4 criteria before and after treatment. The proportion of patients with moderate and severe disease declined, while those classified as having mild disease increased after treatment. (c) Stratified boxplots of IHS4 values before and after treatment across anatomical regions. Median IHS4 scores improved in all regions, with the greatest reductions observed in the chest and face. The *p*‐values indicate within‐group comparisons.

Sixteen of the 17 evaluable patients demonstrated clinical improvement, and nine achieved an IHS4‐55 response. The mean reduction in IHS4 score was 4.7 ± 2.5. Treatment responses varied by anatomical site, with the highest success rates observed in the face, chest, and gluteal regions (Figure 1). A visual summary of anatomical distribution, responder rates, and mean IHS4 reductions is presented in Figure 2. The face was the most frequently treated site (*n* = 6), with a mean IHS4 reduction of 6.3, and three patients achieving IHS4‐55. The chest and gluteal regions were treated in four patients each, with three and two patients, respectively, reaching the IHS4‐55 threshold. The groin, although commonly affected, showed greater variability in response, with only one of the four patients achieving IHS4‐55 and a mean reduction of 2.8. A single patient treated at the nape demonstrated a marked improvement, achieving IHS4‐55 with an eight‐point reduction.

### 3.3 Ultrasound Findings

**FIGURE 3:**
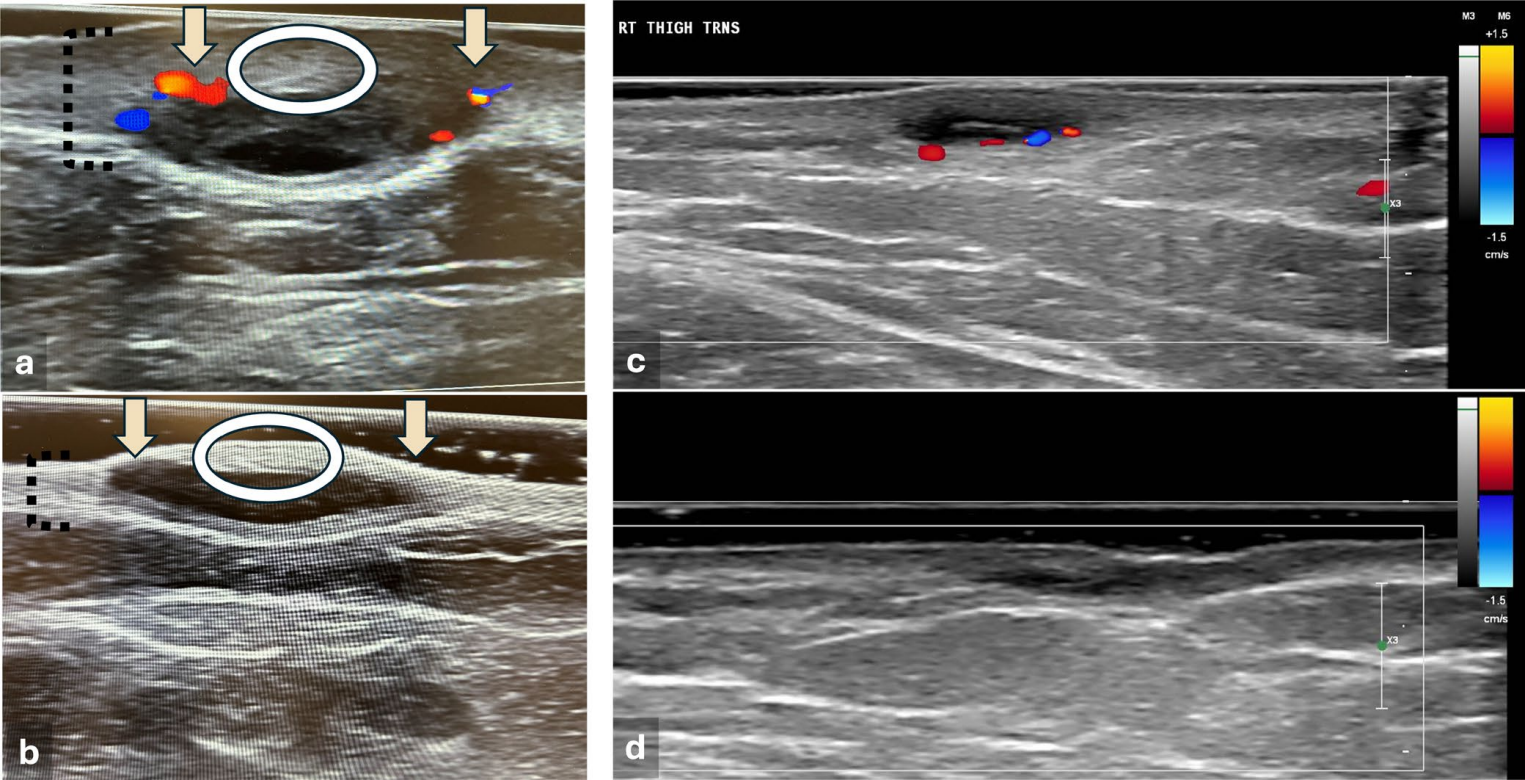
High‐frequency ultrasound evaluation before and after fractional microneedling radiofrequency (RF) treatment. (a–b) HFUS images of a dermal tunnel on the buttocks before treatment (a) and after five sessions of fractional microneedling RF (b), demonstrating reduced dermal thickness (dashed lines), increased echogenicity (circle), decreased tunnel size, and loss of vascular flow (arrows), consistent with resolution of active inflammation. (c–d) Type A dermal tunnel in the thigh with baseline findings of dermal thickening, reduced echogenicity, and a central hyperechoic line consistent with retained hair follicles. Color Doppler revealing hypervascularity at the tunnel base (c). After two treatment sessions (d), there is marked reduction in tunnel size, increased dermal echogenicity and thickness, and complete loss of vascular flow on Doppler imaging.

Due to reimbursement limitations and variable insurance coverage, HFUS follow‐up was performed in only a limited number of cases. In three illustrative patients, imaging confirmed reduced dermal thickness, increased echogenicity, decreased sinus tract size, and diminished Doppler signal post‐treatment findings consistent with reduced inflammation and lower disease activity [13] (Figure 3).

### 3.4 Serious Adverse Events and Discontinuation

Eight patients discontinued treatment after the initial session. One of them who had severe, active disease developed Henoch‐Schönlein purpura vasculitis with joint involvement during the treatment period and subsequently discontinued both follow‐up and therapy. Three patients cited procedural pain as the primary reason for early discontinuation. One patient developed a post‐treatment abscess in the chest requiring hospitalization and intravenous antibiotics, representing the only serious adverse event observed among the study participants. The remaining three patients were either lost to follow‐up or unable to continue due to logistical constraints, such as scheduling difficulties or insurance‐related delays. No long‐term complications, systemic flares, or pigmentary changes were observed in the treated areas during the follow‐up period, which ranged from 3 months after the last treatment session to up to 2 years, depending upon the timing of each patient's treatment within the study timeframe.

### 3.5 Expected Downtime and Postprocedure Care

Transient postprocedural effects were common and expected. All patients reported localized erythema, swelling, mild pain, scabbing, and tenderness over active lesions, typically resolving within 10–14 days. To reduce the risk of bacterial superinfection, all patients were instructed to apply a topical combination antibiotic‐steroid ointment (Betacorten‐G) twice daily for 5–7 days and to cleanse the treated area with antiseptic soap (Septal Scrub) three times weekly.

### 3.6 Patient‐Reported Outcomes

**FIGURE 4:**
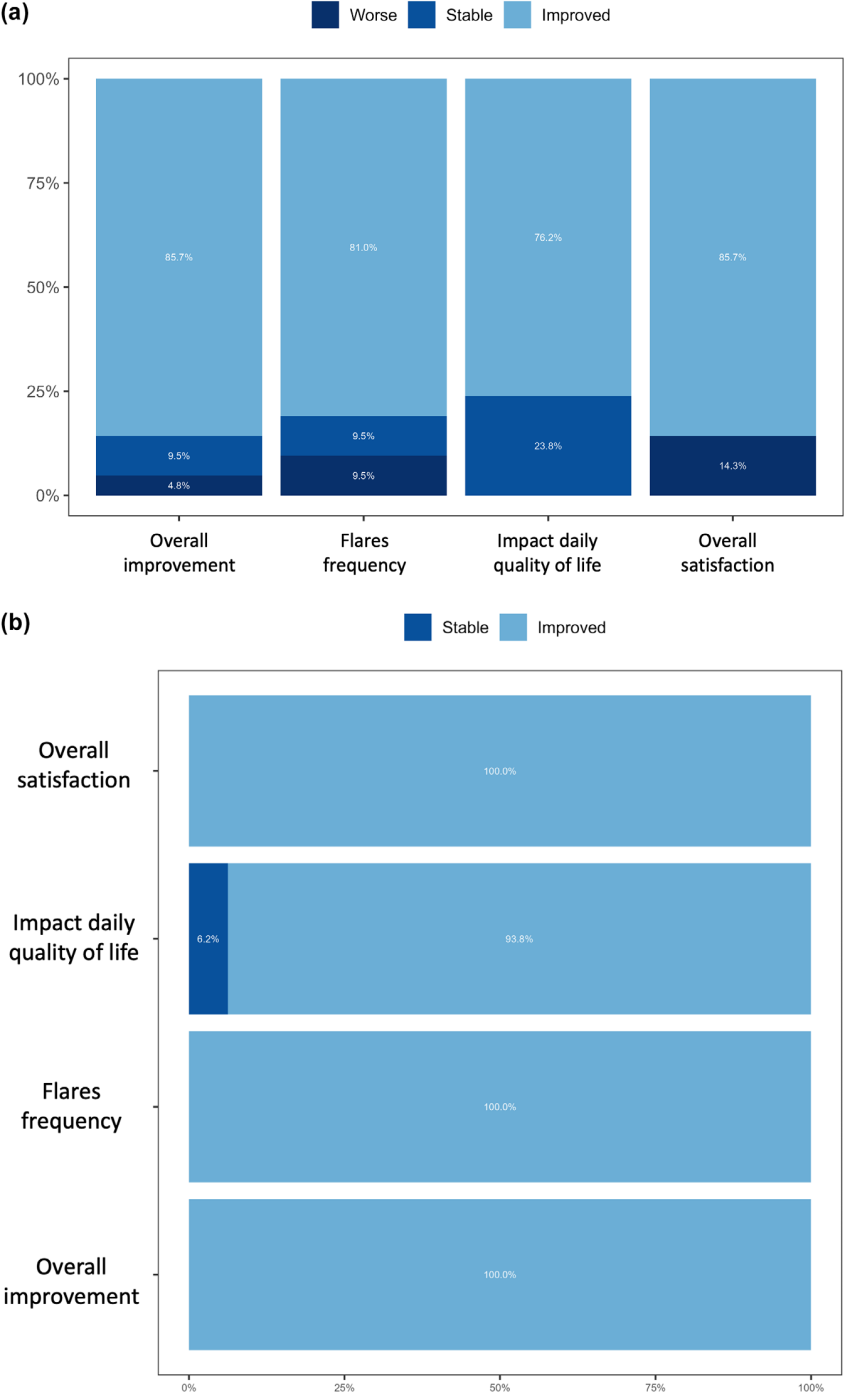
Patient‐reported outcomes following treatment. Stacked bar charts showing patient responses to a structured Patient Global Impression of Change (PGIC)‐based questionnaire across four domains: Overall improvement, flare frequency, daily functioning, and overall satisfaction. (a) Responses from the full cohort of 19 patients (b) Responses from the subgroup of patients who completed two or more treatment sessions. The responses are grouped into three categories: “Worse,” “Stable,” and “Improved,” demonstrating the high rate of favorable outcomes, especially among those who underwent multiple sessions.

Nineteen patients completed the PGIC‐based satisfaction questionnaire. On average, they reported moderate improvement in their overall condition following treatment, with a mean score of 2.4 (mode: 2) on a seven‐point scale where 1 indicated “very much improved.” The perceived frequency of new flares or lesions showed a mean score of 1.95 (mode: 1), suggesting a trend toward reduced disease activity. Daily functioning was reported to have improved, with a mean score of 1.95 (mode: 2), and overall treatment satisfaction was high, with a mean score of 2.0 (mode: 2) on a scale where 1 represented “very satisfied.” Several patients noted that while treatment was painful, it was perceived as worthwhile. The most significant clinical improvements were reported after the third session (Figure 4).

## 4 Discussion

RF technology has gained increasing attention in dermatology for its ability to deliver controlled thermal energy into the dermis via electrical resistance independent of melanin or vascular chromophores. This nonablative modality stimulates collagen remodeling, modulates inflammation, and targets the pilosebaceous unit, all of which are mechanisms that have shown clinical benefit in several chronic follicular and inflammatory dermatoses, including acne vulgaris and rosacea [6]. RF treatments have also demonstrated bacterial load reduction in preclinical models [14]. Given the central role of follicular occlusion, dysregulated inflammation, progressive fibrosis, and microbial imbalance in HS, there is growing interest in RF‐based interventions as potential adjunctive or standalone therapies for this condition.

Clinical evidence supporting the use of RF in HS has emerged from studies on LAight therapy, which combines bipolar RF with intense pulsed light (IPL). This approach demonstrated significant improvement in two multicenter randomized controlled trials involving patients with Hurley stage I and II disease, and was shown to maintain their long‐term remission and enhance quality of life [15, 16]. These findings led to the incorporation of RF‐IPL therapy into the 2024 German S2k guidelines as a recommended treatment option for mild‐to‐moderate HS [8]. Further innovation has led to the development of FMR, a more targeted and penetrative advancement of RF technology. These devices deliver bipolar RF energy through fully insulated microneedles to variable depths, enabling precision thermal injury within the dermis while sparing the epidermis. This approach has already demonstrated efficacy in both follicular and inflammatory dermatoses, including acne vulgaris and rosacea [5, 6], and new potential indications for this technology are continuing to emerge.

A split‐body pilot study by Yang et al. [7] evaluated FMR in 10 patients with Hurley II disease. The treated sites showed significantly improved HS‐PGA and modified Sartorius scores compared to control sites, along with marked histologic reductions of cytokines, such as TNF‐α, IL‐17, IL‐8, and TGF‐β1, and upregulation of collagen I expression. These molecular and clinical changes strongly suggest an attenuation of inflammation and early tissue remodeling. A recent systematic review and meta‐analysis confirmed that RF therapies, including FMR, consistently improve HS clinical severity scores, pain, and DLQI, with a favorable safety profile across studies [17].

Despite growing interest in RF‐based therapies for HS, existing clinical evidence remains limited in scope, population diversity, methodological inconsistency, as well as addressing only typical (axillary and groin) regions [17]. Moreover, the use of heterogeneous outcome measures—many of which lack sensitivity to dynamic changes in inflammatory activity—has hindered direct comparison across studies and limited broader clinical translation.

Building on the findings of prior investigations described above, our current study aimed to assess the clinical utility of FMR in a real‐world cohort that included treatment‐resistant patients from a demographically and clinically diverse population, with lesions in previously underrepresented and difficult‐to‐treat anatomical sites, such as the face, scalp, chest, and intermammary folds. We evaluated the therapeutic outcomes by using IHS4 and IHS4‐55, a dynamic scoring system that provides a weighted assessment of nodules, abscesses, and draining tunnels, and one that is now considered the preferred outcome measure for clinical trials and routine monitoring according to the 2024 German S2k guidelines [8, 9, 18]. Our aim was to offer a standardized tracking of changes in disease activity and treatment outcomes over time.

FMR treatment demonstrated clinically meaningful outcomes in treatment‐resistant HS, with 16 of the 17 patients who completed at least two treatment sessions (94.1%) showing clinical improvement, and nine (52.9%) achieving an IHS4‐55 response. These results are particularly noteworthy given our cohort's therapy‐resistant nature and the inclusion of surgically complex anatomical sites. We also observed variation in treatment response by anatomical location. The face was the most frequently treated site (*n* = 6), demonstrating both a substantial mean IHS4 reduction (6.3 points) and a 50% IHS4‐55 response rate, highlighting the potential utility of FMR in cosmetically sensitive areas. The chest and gluteal regions also showed strong clinical outcomes, with three of four and two of four patients, respectively, achieving IHS4‐55. In contrast, groin lesions, although commonly affected in HS, demonstrated greater variability in response: only one of four patients reached IHS4‐55, with a modest mean reduction of 2.8 points. These findings suggest that anatomical factors, such as skin thickness, follicular density, and vascular supply may influence treatment efficacy.

Treatment was generally well tolerated. All patients reported transient postprocedural effects, including localized erythema, swelling, scabbing, and pain, typically resolving within 5–10 days. Short‐lived tenderness over active lesions was also commonly described, subsiding within 10–14 days. Nevertheless, several patients reported significant pain during the treatment, which led to early discontinuation in 3 of 25 cases (12%). These instances occurred primarily in sensitive areas such as the groin, thigh, and inframammary folds, highlighting the potential need for tailored anesthesia protocols and enhanced pain management strategies in these regions.

Patient‐reported outcomes supported the observed clinical improvements: in a structured PGIC‐based questionnaire completed by 19 patients, 86.4% (*n* = 16) reported moderate or marked improvement. Among the 13 patients who received at least two treatment sessions, all reported improvement in general symptoms and flare frequency, and 84.6% noted better daily functioning, suggesting a cumulative therapeutic benefit with repeated sessions. Despite procedural discomfort, all 13 patients in this subgroup felt that the therapeutic benefits justified continued treatment. These findings underscore not only the clinical efficacy of FMR, but also its perceived value and acceptability among patients with recalcitrant HS, particularly when administered over multiple sessions.

Based on these observations, we recommend that medical teams provide clear preprocedural counseling regarding expected discomfort and recovery. While topical anesthesia was adequate for many patients, tumescent or regional anesthesia should be considered for areas associated with heightened sensitivity or procedural intolerance. Following treatment, a standardized regimen of topical corticosteroid‐antibiotic ointment (Betacorten‐G) and antiseptic cleansing (Septal Scrub) was prescribed to reduce inflammation and minimize the risk of secondary infection. Both clinical response and patient‐reported outcomes in our cohort generally improved after the second or third session, highlighting the need for setting realistic expectations and encouraging treatment adherence. In this context, reassurance based on early objective improvements and favorable safety data may enhance patient engagement and support long‐term treatment success.

This study is limited by its retrospective design, small sample size, lack of a control group, and relatively short follow‐up duration. The modest number of patients enrolled over a 2‐year period reflects the real‐world complexity of offering a novel, nonuniversally approved treatment within a public healthcare system. Although FMR was offered to all eligible patients as an alternative therapeutic option, most of them were required to obtain insurance authorization for the payment of each session. Delays or denials in coverage limited both treatment initiation and continuity, and only few patients elected to self‐fund therapy. Treatment parameters (depth, energy, mode) were selected based upon clinical judgment, lesion location, existing literature, and pathophysiologic characteristics of HS. In the absence of a standardized protocol, this individualized approach, while pragmatic, limits reproducibility and cross‐study comparability. Treatment intervals also varied: while a 4–6 week interval was generally targeted during active disease, with longer spacing during maintenance, real‐world factors, such as insurance delays, out‐of‐pocket costs, and healthcare system‐related disruptions ‐ contributed to inconsistency in treatment scheduling.

Despite these limitations, the findings of this study offer valuable insights into the feasibility, safety, and patient‐reported outcomes of FMR in a treatment‐resistant HS population under real‐world conditions. To the best of our knowledge, this represents the largest cohort to date among whom FMR was evaluated for HS. The study population was diverse in terms of age, sex, and skin type, and included patients with lesions across a broad anatomical spectrum. Clinical outcomes were assessed by means of standardized and validated scoring systems, providing robust and dynamic measures of inflammatory activity and treatment response. In addition, HFUS imaging provided objective validation of clinical improvements, demonstrating reduced dermal thickness, resolution of hypoechoic sinus tracts, and decreased vascular flow post‐treatment all in support of FMR's anti‐inflammatory effects and highlighting its utility as a monitoring tool.

Our findings demonstrated clinically meaningful improvement in the majority of patients, reinforcing the potential role of FMR in the therapeutic arsenal for HS. The observed 53% IHS4‐55 response rate compares favorably with established therapies, approaching the efficacy seen with some biologic agents in clinical trials [19] and supports FMR as a viable therapeutic option for patients with persistent or refractory disease, including those seeking adjunctive, lesion‐targeted interventions to optimize outcomes. Its repeatability, anatomical precision, and lack of systemic adverse effects position FMR as especially suitable for long‐term management.

Taken together, these results provide a compelling rationale for further exploration of FMR in the setting of HS. Future prospective studies, including randomized controlled trials, are warranted to refine treatment parameters, optimize session intervals for induction and maintenance phases, determine appropriate treatment durations, and tailor treatment protocols according to disease severity, anatomical location, and patient tolerability. Additional efforts should also focus on improving pain management strategies and clarifying the role of FMR within the evolving therapeutic landscape for HS.

## Author Contributions

A.S. supervised data collection and analysis, interpreted clinical data, coordinated project administration and IRB approval, and drafted and revised the manuscript. E.T. assisted in patient follow‐up and documentation, performed and evaluated ultrasound examinations, interpreted imaging findings, and contributed to manuscript editing. D.B., M.A., A.L., and W.S., contributed to the literature review, and assisted in manuscript editing. A.H. participated in patient evaluation and follow‐up, contributed to clinical interpretation and discussion, and reviewed manuscript drafts. O.A. supervised development of the treatment protocol, contributed to study design and critical revision. All authors have read and approved the final manuscript.

## Funding

The authors have nothing to report.

## Ethics Statement

The study protocol was approved by the local institutional review board (#0452‐24). Patients provided written informed consent for the use of their anonymized photographs for research and publication purposes.

## Conflicts of Interest

Prof. Ofir Artzi serves occasionally as a key opinion leader for InMode Ltd. and holds a small, noninfluential personal shareholding in the company. All other authors declare no conflicts of interest.

## Supporting information


**Table S1:** Patient global impression of change (PGIC) questionnaire
Structured follow‐up questionnaire used to assess patient‐reported outcomes after FMR treatment for hidradenitis suppurativa. The form included global impression of change, pain levels, flare frequency, daily functioning, and overall satisfaction
**Table S2:** FMR treatment parameters by anatomical region
Detailed treatment protocols used for fractional microneedling radiofrequency (FMR) across different anatomical regions. The general protocol is provided along with modifications made for sensitive areas such as the groin and face. Parameters include depth, mode, energy level, number of stacks, and treatment passes.

## Data Availability

The data that support the findings of this study are available on request from the corresponding author. The data are not publicly available due to privacy or ethics restrictions.
