# Supplementary material for: Fractional Microneedling Radiofrequency for Hidradenitis Suppurativa: A Real‐World Retrospective Study Demonstrating Clinical Efficacy and Safety Across Diverse Anatomical Sites
Source: J Cosmet Dermatol. 2026 Feb 24;25(3):e70748. doi: 10.1111/jocd.70748 (PMC12930327; doi:10.1111/jocd.70748)
Supplement: Supplementary file 1 — Table S1: Patient global impression of change (PGIC) questionnaire Structured follow‐up questionnaire used to assess patient‐reported outcomes after FMR treatment for hidradenitis suppurativa. The form included global impression of change, pain levels, flare frequency, daily functioning, and overall satisfaction Table S2: FMR treatment parameters by anatomical region Detailed treatment protocols used for fractional microneedling radiofrequency (FMR) across different anatomical regions. The general protocol is provided along with modifications made for sensitive areas such as the groin and face. Parameters include depth, mode, energy level, number of stacks, and treatment passes. [file JOCD-25-e70748-s001.docx]

**Supplementary Table 1. Patient Global Impression of Change (PGIC) Questionnaire**

Structured follow-up questionnaire used to assess patient-reported outcomes after FMR treatment for hidradenitis suppurativa. The form included global impression of change, pain levels, flare frequency, daily functioning, and overall satisfaction

1. **Global Impression of Change (PGIC)**

Thinking about your Hidradenitis Suppurativa condition overall, how do you feel it has changed since your treatment?

| **Response Options** |
| --- |
| 1 - Very much improved |
| 2 - Much improved |
| 3 - Minimally improved |
| 4 - No change |
| 5 - Minimally worse |
| 6 - Much worse |
| 7 - Very much worse |

1. **Pain Assessment**

How would you describe pain from your HS since treatment?

| **Response Options** |
| --- |
| Much better |
| Somewhat better |
| About the same |
| Somewhat worse |
| Much worse |

1. **Lesion/Flare Frequency**

How has the frequency of flare-ups or new lesions changed since your treatment?

| **Response Options** |
| --- |
| Much less frequent |
| Somewhat less frequent |
| About the same |
| Somewhat more frequent |
| Much more frequent |

1. **Impact on Daily Activities**

How has your condition affected your daily activities?

| **Response Options** |
| --- |
| Much easier to do things |
| Somewhat easier |
| No change |
| Somewhat harder |
| Much harder |

1. **Overall Satisfaction**

Overall, how satisfied are you with the results of your treatment?

| **Response Options** |
| --- |
| Very satisfied |
| Satisfied |
| Neutral |
| Dissatisfied |
| Very dissatisfied |

**Supplementary Table 2. FMR Treatment Parameters by Anatomical Region**

The general treatment protocol consisted of fractional microneedling radiofrequency (FMR) therapy using the Morpheus8 Body 3D tip (InMode). Treatment parameters varied by anatomical region and were adjusted based on anatomical considerations, pain sensitivity, and lesion characteristics. Post-treatment care included topical Betacorten-G ointment, containing betamethasone valerate (0.1%) and gentamicin sulfate (0.1%), applied twice daily for 5–7 days.

Detailed treatment protocols used for fractional microneedling radiofrequency (FMR) across different anatomical regions. The general protocol is provided along with modifications made for sensitive areas such as the groin and face. Parameters include depth, mode, energy level, number of stacks, and treatment passes.

## General Protocol

| **Depth (mm)** | **Mode** | **Energy** | **Stacks** | **Passes** |
| --- | --- | --- | --- | --- |
| 7 | Fixed | 60 | 3 | 2 |
| 6 | Fixed | 60 | 3 | 2 |
| 5 | Fixed | 40–45 | 3 | 1 |
| 4 | Fixed | 35–40 | 3 | 1 |
| 3 | Cycle | 35–40 | 3 | 1 |

## Groin (Adjusted Protocol)

| **Depth (mm)** | **Mode** | **Energy** | **Stacks** | **Passes** |
| --- | --- | --- | --- | --- |
| 4 | Fixed | 35 | 3 | 1 |
| 3 | Fixed | 30 | 3 | 1 |
| 2 | Fixed | 20 | 2 | 2 |

## Face (Adjusted Protocol)

| **Depth (mm)** | **Mode** | **Energy** | **Stacks** | **Passes** |
| --- | --- | --- | --- | --- |
| 5 | Fixed | 50 | 3 | 2 |
| 4 | Fixed | 40 | 3 | 1 |
| 3 | Fixed | 30 | 3 | 1 |
| 2 | Fixed | 20 | 2 | 1 |
